# Ideal patients for liver resection in Barcelona Clinic Liver Cancer or Hong Kong Liver clinic systems for hepatocellular carcinoma: Conservative or aggressive?

**DOI:** 10.3389/fmed.2022.977135

**Published:** 2022-10-14

**Authors:** Jun-Xiang Li, Peng Zhou, De-Hua Chang, Yao Tong, Yan Bao, Yu-Dong Xiao, Shi Zhou, Wen-Wu Cai

**Affiliations:** ^1^Department of Interventional Radiology, Guizhou Medical University Affiliated Cancer Hospital, Guiyang, China; ^2^Department of Pathology, The Second Xiangya Hospital of Central South University, Changsha, China; ^3^Department of Diagnostic and Interventional Radiology, University Hospital Heidelberg, Heidelberg, Germany; ^4^Department of Radiology, The Second Xiangya Hospital of Central South University, Changsha, China; ^5^Department of Radiology, The Affiliated Hospital of Guizhou Medical University, Guiyang, China; ^6^Department of Liver Surgery, The Second Xiangya Hospital of Central South University, Changsha, China

**Keywords:** hepatocellular carcinoma, liver resection, ideal patients, prognosis, comparison

## Abstract

**Background:**

Both the Barcelona Clinic Liver Cancer (BCLC) staging and the Hong Kong Liver Cancer (HKLC) staging have their own definitions of ideal patients for liver resection (IPLR) in hepatocellular carcinoma (HCC). This study aimed to compare the prognosis of IPLRs between the BCLC and HKLC staging systems, and to identify patients who may benefit from liver resection (LR) in the HKLC staging but beyond the BCLC staging.

**Methods:**

This retrospective study evaluated 1,296 consecutive patients with HCC who underwent LR between August 2013 and April 2021 (457 patients and 1,046 patients were IPLR according to the BCLC and HKLC staging systems, respectively). Overall survival (OS) was compared between the two groups. To assess potential benefit of LR for IPLR in the HKLC staging but beyond the BCLC staging, univariate and multivariate Cox regression analysis was performed to determine prognostic factors of OS, and prognostic stratification was performed based on the selected prognostic factors. The IPLRs in the HKLC staging but beyond the BCLC staging were divided into subgroups according to the prognostic stratification and separately compared with the IPLRs in the BCLC staging.

**Results:**

OS was different between the two staging systems (*P* = 0.011). All the 457 IPLRs in the BCLC staging were also the IPLRs in the HKLC staging. Diameter of the largest tumor5 cm (HR = 1.58; 95% CI: 1.18–2.10; *P* = 0.002) and liver cirrhosis (HR = 1.61; 95% CI: 1.19–2.20; *P* = 0.002) were risk factors for poor OS in IPLRs in the HKLC staging but beyond the BCLC staging; hence, patients were divided into the low-risk (*n* = 104), intermediate-risk (*n* = 369), and high-risk groups (*n* = 116) accordingly. There was no difference in OS between patients in the BCLC staging and patients in low-risk group (*P* = 0.996). However, OS was significantly different between patients in the BCLC staging and those in intermediate-risk (*P* = 0.003) and high-risk groups (*P* < 0.001).

**Conclusion:**

IPLRs in the BCLC staging system have better prognosis. However, IPLRs in the HKLC staging system but beyond the BCLC staging may have equivalent prognosis to IPLRs in the BCLC staging if the tumor size is ≤ 5 cm and liver cirrhosis is absent.

## Introduction

Hepatocellular carcinoma (HCC) is the most common primary liver cancer worldwide, with high morbidity and mortality rates (1). Several guidelines suggest liver resection (LR) as a main curative treatment option for HCC (2, 3). However, the prognosis of HCC after LR differs significantly owing to the heterogeneity of the HCC population (4). Several scoring and staging systems have been developed to stratify the prognosis of patients with HCC (5–7), with the Barcelona Clinic Liver Cancer (BCLC) staging system being the most popular treatment guideline in Western countries (8). The BCLC staging system has been endorsed by both the European Association for the Study of the Liver and the American Association for the Study of Liver Diseases. However, the BCLC staging system is not widely accepted in Asia, because the cause of HCC in the east is different to that in west. The Hong Kong Liver Cancer (HKLC) staging system was developed in Asia in 2014 (9). Like the BCLC staging system, the HKLC staging system also incorporates performance status (PS), liver function, and tumor burden and links prognostic classification to treatment indications. Both staging systems have proposed ideal patients for LR (IPLR), and the definitions of IPLR between two staging systems are rather different. Several studies have compared the prognostic predictive value of these two staging systems for HCC (10–13). However, the prognosis of IPLRs between these two staging systems still remain unknown. In addition, the definition in IPLR in the BCLC staging system is usually considered to be conservative while that in the HKLC staging is considered to be aggressive. Therefore, it needs to be determined whether the IPLRs in the HKLC staging system but beyond the BCLC staging system may potentially benefit from the LR. As such, this study aimed to (1) compare the difference in overall survival (OS) of IPLRs between the BCLC and the HKLC staging; (2) compare the difference in OS between IPLRs in the BCLC staging system and those in the HKLC staging system but beyond the BCLC staging system; and (3) investigate the risk factors of poor OS and identify potential patients who may benefit from LR in IPLRs in the HKLC staging system but beyond the BCLC staging system.

## Materials and methods

### Study design and population

This retrospective study evaluated consecutive patients with pathologically proven HCC who underwent LR in three tertiary referral hospitals between August 2013 and April 2021. The exclusion criteria were as follows: (1) unavailability of follow-up data, (2) unavailability of baseline imaging information within 1 month preoperatively, (3) incomplete resection of all tumor nodules, (4) unavailability of baseline laboratory information within 1 week preoperatively, and (5) history of systemic therapy or locoregional therapy prior to LR.

This study was approved by the institutional review board of our hospitals and was performed according to the Declaration of Helsinki. The institution review board waived the requirement for written informed consent owing to the retrospective nature of the study.

### Data collection

Data on clinical variables, including age, sex, etiology of underlying liver diseases, and Eastern Cooperative Oncology Group (ECOG) Performance Status score, were collected. Imaging parameters included the diameter of the largest tumor, number of tumors, ascites, macroscopic vascular invasion, liver cirrhosis, and portal hypertension. Liver cirrhosis was diagnosed by pathology, which was defined as an advanced form of progressive hepatic fibrosis with distortion of the hepatic architecture and regenerative nodule formation. In our institutions, direct measurement of portal venous pressure was not routinely performed; therefore, portal hypertension was defined as either splenomegaly, ascites, or varices on imaging or a platelet count < 100 × 109. Laboratory parameters included neutrophil count, lymphocyte count, platelet count, serum albumin, total bilirubin, alanine aminotransferase (ALT), aspartate aminotransferase (AST), creatinine, international normalized ratio, and alpha-fetoprotein (AFP).

### Follow-up protocol and outcome measures

Patients were observed during follow-up every 2–3 months postoperatively and at least every 6 months thereafter. Follow-up comprised routine measurements of serum AFP levels and liver function and liver ultrasonography to monitor for recurrence. If recurrence was suspected, contrast-enhanced computed tomography or magnetic resonance imaging was performed to verify the recurrence. If tumor recurrence occurred, the choice of treatment modality was based on the tumor site, liver function, and general condition of the patient. The primary outcome measure was OS, defined as the time interval between the date of surgery and the date of any-cause death. The last follow-up was defined as the time of the last telephone interview (March 2022) or the last visit to the hospital if a telephone interview was unavailable.

### Statistical analysis

Continuous variables are reported as medians and interquartile ranges (IQR) (age is presented as mean and standard deviation) and compared using the Mann-Whitney *U*-test, Student’s *t*-test or Kruskal-Wallis test, or One-way ANOVA (if appropriate). Meanwhile, categorical variables are presented as frequencies and percentages and compared using the chi-square test or Fisher’s exact test (if appropriate). The difference in OS between IPLRs in the BCLC staging system and those in the HKLC staging system was estimated using the Kaplan-Meier method and compared using log-rank tests. Similarly, the difference in OS was also compared between IPLRs in the BCLC staging and those in the HKLC staging system but beyond the BCLC staging system. The risk factors for poor OS in IPLRs in the HKLC staging system but beyond the BCLC staging system were determined. Differences in sex, age, diameter of the largest tumor, number of tumors, etiology of underlying liver diseases, liver cirrhosis, albumin, total bilirubin, ALT, AST, AFP, portal hypertension, macroscopic vascular invasion, and creatinine according to OS were compared using univariate Cox regression analysis. The optimal cut-off values for serum albumin (≥ 35/ < 35 g/L), total bilirubin (≤ 1.0/ > 1.0 mg/dL), ALT (≤ 40/ > 40 IU/L), AST (≤ 40/ > 40 IU/L), and creatinine (≤ 1.2/ > 1.2 mg/dL) were determined based on the upper or lower limits of the normal range. For the diameter of the largest tumor, number of tumors, and AFP level, the optimal cut-off values were 5 cm, single/2–3/ > 3, and 200 ng/mL, respectively.

Significant variables in the univariate analysis (i.e., those with *P*-values < 0.05) were entered in a multivariate backward Cox regression analysis. The prognostic stratification for IPLRs in the HKLC staging system but beyond the BCLC staging system was performed. To identify patients who may benefit from LR in IPLRs in the HKLC staging system but beyond the BCLC staging system, the prognostic stratification was performed by assigning exact points for each variable in proportion to the beta coefficients in the final Cox regression model with statistically significant predictors. The total points for each patient were the sum of the points of the identified parameters. The patients were then stratified into low, intermediate, and high risk of death groups using a set of clinical factors that had the best prognostic performance in the multivariate Cox regression analysis. The differences in the OS of each group were compared with those of patients in the BCLC staging system. All statistical analyses were performed using SPSS version 20 (IBM Corp., Armonk, NY, USA) or R software (version 4.0.2).^1^ All tests were two sided, and *P*-values < 0.05 were considered statistically significant.

## Results

### Baseline characteristics of the entire study population

**FIGURE 1:**
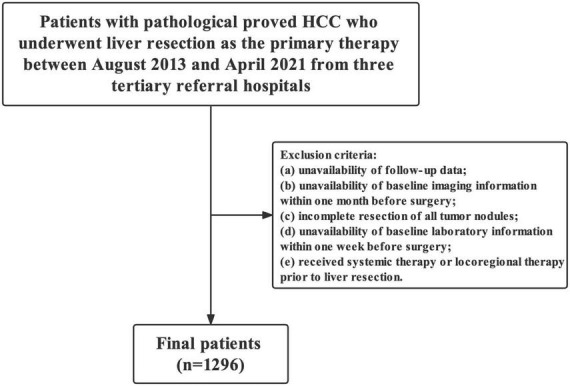
Flowchart of the study.

**FIGURE 2:**
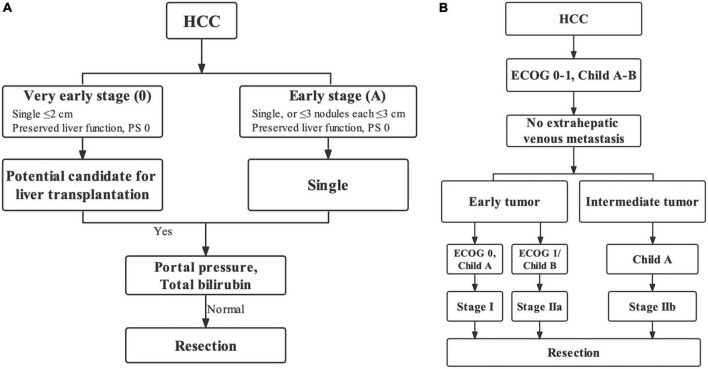
Ideal patients for liver resection (IPLR) in the BCLC staging system **(A)** and HKLC staging system **(B)**.

Overall, 457 (35.3%, 457/1,296) and 1,046 (80.7%, 1,046/1,296) patients were IPLR according to the 2022 version of the BCLC and HKLC staging systems, respectively. The patient selection flowchart is shown in Figure 1. The definitions of IPLRs in the BCLC and HKLC staging systems are illustrated in Figure 2. The median follow-up duration of the entire study population was 22.8 months (IQR: 10.0–41.0 months). By the end of the last follow-up, telephone interviews were successfully performed for 1,075 patients (82.9%, 1,075/1,296), and 464 patients died (35.8%, 464/1,296). The median OS in the entire study population was 60.6 months (95% CI: 51.7–69.5), and the 1-, 2-, 3-, 4-, and 5-year OS rates were 81.9, 70.4, 61.9, 55.8, and 49.9%, respectively. The baseline characteristics of the study population are summarized in Table 1.

**TABLE 1:** Baseline characteristics of the entire study population.

Baseline characteristics	Overall (*n* = 1,296)	Hospital A (*n* = 1,017)	Hospital B (*n* = 160)	Hospital C (*n* = 119)	*P*-value
Age (years, mean, SD)	52.9 ± 12.0	52.5 ± 11.5	55.7 ± 13.5	52.5 ± 13.0	0.007
Gender (N,%)					0.276
Male	1,100 (84.9)	869 (85.4)	129 (80.6)	102 (85.7)	
Female	196 (15.1)	148 (14.6)	311 (19.4)	17 (14.3)	
Etiology of underlying liver diseases (N,%)					< 0.001
None	264 (20.4)	189 (18.6)	46 (28.8)	29 (24.4)	
HBV	901 (69.5)	704 (69.2)	110 (68.8)	87 (73.1)	
HCV	26 (2.0)	22 (2.2)	1 (0.6)	3 (2.5)	
Alcohol	30 (2.3)	29 (2.9)	1 (0.6)	0 (0.0)	
Mixed	75 (5.8)	73 (7.2)	2 (1.2)	0 (0.0)	
ECOG ps (N,%)					0.945
0	1,164 (89.8)	916 (90.1)	142 (88.8)	106 (89.1)	
1	95 (7.3)	72 (7.1)	14 (8.8)	9 (7.6)	
2	37 (2.9)	29 (2.9)	4 (2.5)	4 (3.4)	
Child-pugh class (N,%)					< 0.001
A	1,206 (93.1)	954 (93.8)	149 (93.1)	103 (86.6)	
B	86 (6.6)	62 (6.1)	11 (6.7)	13 (10.9)	
C	4 (0.3)	1 (0.1)	0 (0.0)	3 (2.5)	
Diameter of the largest tumor (mm, median, IQR)	54.0 (33.0–84.0)	55.0 (34.0–85.0)	45.2 (31.2–81.0)	47 (31.5–71.0)	0.032
Number of tumors (N,%)					0.261
1	1,075 (82.9)	838 (82.4)	137 (85.6)	100 (84.0)	
2–3	148 (11.4)	115 (11.3)	20 (12.5)	13 (10.9)	
> 3	73 (5.6)	64 (6.3)	3 (1.9)	6 (5.0)	
Ascites (N,%)					< 0.001
Absence	1,145 (88.3)	921 (90.6)	144 (90.0)	80 (67.2)	
Mild	135 (10.4)	89 (8.8)	15 (9.4)	31 (26.1)	
Moderate-massive	16 (1.2)	7 (0.7)	1 (0.6)	8 (6.7)	
Liver cirrhosis (N,%)					< 0.001
Absence	589 (45.4)	453 (44.5)	104 (65.0)	32 (26.9)	
Presence	707 (54.6)	564 (55.5)	56 (35.0)	87 (73.1)	
Portal hypertension (N,%)					0.006
Absence	829 (64.0)	669 (65.8)	99 (61.9)	61 (51.3)	
Presence	467 (36.0)	348 (34.2)	61 (38.1)	58 (48.8)	
Macroscopic vascular invasion (N,%)					< 0.001
Absence	1,103 (85.1)	843 (82.9)	146 (91.3)	114 (95.8)	
Intrahepatic vascular invasion	174 (13.4)	157 (15.4)	13 (8.1)	4 (3.4)	
Extrahepatic vascular invasion	19 (1.5)	17 (1.7)	1 (0.7)	1 (0.8)	
Neutrophil count (× 10^9^, median, IQR)	3.43 (2.50–4.51)	3.48 (2.58–4.53)	3.05 (2.16–4.00)	3.56 (2.39–4.67)	0.001
Lymphocyte count (× 10^9^, median, IQR)	1.35 (1.03–1.73)	1.36 (1.03–1.71)	1.35 (1.07–1.86)	1.30 (0.93–1.75)	0.233
Platelet count (× 10^9^, median, IQR)	158 (111–214)	159 (114–216)	148 (111–201)	158 (102–215)	0.344
Creatine (mg/dL, median, IQR)	0.79 (0.68–0.90)	0.80 (0.69–0.91)	0.75 (0.63–0.86)	0.75 (0.67–0.88)	< 0.001
Serum albumin (g/L, median, IQR)	38,9 (35.9–41.8)	38.6 (35.8–41.2)	40.3 (36.4–43.5)	41.7 (37.8–44.2)	< 0.001
Total bilirubin (mg/dL, median, IQR)	0.80 (0.59–1.10)	0.80 (0.60–1.10)	0.75 (0.51–1.08)	0.86 (0.61–1.16)	0.024
ALT (IU/L, median, IQR),	33.2 (22.6–52.2)	32.9 (22.4–52.5)	33.2 (22.0–49.5)	37.4 (26.4–53.0)	0.122
AST (IU/L, median, IQR)	38.2 (26.8–55.8)	37.7 (26.6–55.7)	37.2 (26.9–54.4)	42.0 (28.4–58.0)	0.207
INR (median, IQR)	1.03 (0.97–1.11)	1.04 (0.97–1.11)	1.04 (0.97–1.11)	1.01 (0.96–1.08)	0.031
AFP (ng/mL, median, IQR).					0.496
≤200	759 (58.6)	587 (57.7)	99 (61.9)	73 (61.3)	
>200	537 (41.4)	430 (42.3)	61 (38.1)	46 (38.7)	
Follow-up duration (months, median, IQR)	22.8 (10.0–41.0)	23.1 (9.1.5–40.8)	23.0 (14.0–42.3)	20.3 (8.5–42.9)	0.430

SD, standard deviation; IQR, interquartile range; HBV, hepatitis B virus; HCV, hepatitis C virus; ECOG, eastern cooperative oncology group; ALT, alanine aminotransferase; AST, aspartate aminotransferase; INR, international normalized ratio; AFP, alpha-fetoprotein.

### Comparison of overall survival between ideal patients for liver resections in the Barcelona Clinic Liver Cancer staging system and those in the Hong Kong Liver Cancer staging system

**FIGURE 3:**
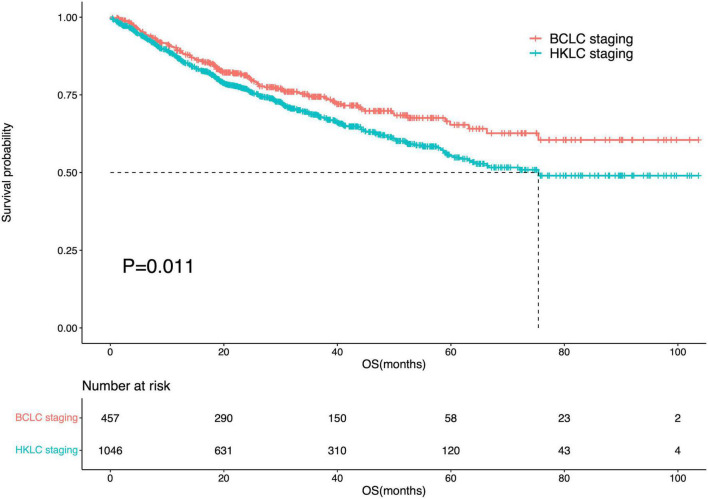
Survival curve of the IPLRs in the BCLC staging system and those in the HKLC staging system.

Among IPLRs in the BCLC staging system, 31 patients had BCLC-0 and 426 patients had BCLC-A disease. Moreover, among IPLRs in the HKLC staging system, 507, 429, and 110 patients had stage I, stage IIa, and stage IIb disease, respectively. The 1-, 2-, 3-, 4-, and 5-year OS rates of IPLRs in the BCLC staging system were 89.0, 81.0, 73.9, 69.1, and 64.1%, respectively. The corresponding percentiles for IPLRs within the HKLC staging were 86.7, 76.7, 68.4, 61.7, and 54.9%, respectively. The median OS was not reached for IPLRs in the BCLC staging system and was 75.4 months (95% CI: N/A) for IPLRs in the HKLC staging system, with a significant difference (*P* = 0.011, Figure 3). The baseline characteristics of IPLRs in the BCLC and HKLC staging systems are summarized in Table 2.

**TABLE 2:** Baseline characteristics of IPLRs in the BCLC and HKLC staging systems.

Baseline characteristics	BCLC staging (*n* = 457)	HKLC staging (*n* = 1,046)	*P*-value
Age (years, mean, SD)	54.1 ± 12.9	53.4 ± 12.0	0.274
Gender (N,%)			0.477
Male	380 (83.2)	885 (84.6)	
Female	77 (16.9)	161 (15.4)	
Etiology of underlying liver diseases (N,%)			0.034
None	126 (27.6)	218 (20.8)	
HBV	286 (62.6)	729 (69.7)	
HCV	5 (1.1)	19 (1.8)	
Alcohol	9 (2.0)	21 (2.1)	
Mixed	31 (6.8)	59 (5.6)	
ECOG ps (N,%)			<0.001
0	457 (100)	1,007 (96.3)	
1	0 (0.0)	39 (3.7)	
2	0 (0.0)	0 (0.0)	
Child-pugh class (N,%)			<0.001
A	456 (99.8)	1,011 (96.7)	
B	1 (0.2)	35 (3.3)	
Diameter of the largest tumor (mm, median, IQR)	52.0 (34.0–80.0)	46.0 (30.0–71.0)	0.002
Number of tumors (N,%)			<0.001
1	457 (100)	916 (87.6)	
2–3	0 (0.0)	118 (11.3)	
> 3	0 (0.0)	12 (1.1)	
Ascites (N,%)			<0.001
Absence	457 (100)	943 (90.2)	
Mild	0 (0.0)	96 (9.2)	
Moderate-massive	0 (0.0)	7 (0.7)	
Liver cirrhosis (N,%)			<0.001
Absence	279 (61.1)	490 (46.8)	
Presence	178 (38.9)	556 (53.2)	
Portal hypertension (N,%)			<0.001
Absence	457 (100)	683 (65.3)	
Presence	0 (0.0)	363 (34.7)	
Macroscopic vascular invasion (N,%)			<0.001
Absence	457 (100)	1,028 (98.3)	
Intrahepatic vascular invasion	0 (0.0)	18 (1.7)	
extrahepatic vascular invasion	0 (0.0)	0 (0.0)	
Neutrophil count (× 10^9^, median, IQR)	3.63 (2.87–4.51)	3.38 (2.41–4.38)	<0.001
Lymphocyte count (× 10^9^, median, IQR)	1.52 (1.20–1.89)	1.37 (1.05–1.77)	<0.001
Platelet count (× 10^9^, median, IQR)	188 (147–235)	156 (108–212)	<0.001
Creatine (mg/dL, median, IQR)	0.79 (0.69–0.91)	0.79 (0.68–0.90)	0.612
Serum albumin (g/L, median, IQR)	39.6 (37.1–42.4)	39.2 (36.4–42.2)	0.100
Total bilirubin (mg/dL, median, IQR)	0.64 (0.50–0.79)	0.78 (0.59–1.07)	<0.001
ALT (IU/L, median, IQR),	30.0 (19.6–45.3)	32.3 (21.6–49.2)	0.011
AST (IU/L, median, IQR)	32.0 (23.7–44.9)	35.4 (26.0–50.3)	<0.001
INR (median, IQR)	1.01 (0.95–1.07)	1.03 (0.97–1.11)	<0.001
AFP (ng/mL, N,%).			0.412
≤200	295 (64.6)	652 (62.3)	
>200	162 (35.4)	394 (37.7)	
OS (months, median, 95%CI)	Not reached	75.4 (N/A)	0.011

IPLR, ideal patients for liver resection; BCLC, Barcelona Clinic Liver Cancer; HKLC, the Hong Kong Liver Cancer; SD, standard deviation; IQR, interquartile range; HBV, hepatitis B virus; HCV, hepatitis C virus; ECOG, eastern cooperative oncology group; ALT, alanine aminotransferase; AST, aspartate aminotransferase; INR, international normalized ratio; AFP, alpha-fetoprotein; OS, overall survival; CI, confidence interval.

### Comparison of overall survival between ideal patients for liver resections in the Barcelona Clinic Liver Cancer staging system and those in the Hong Kong Liver Cancer staging but beyond the Barcelona Clinic Liver Cancer staging system

**FIGURE 4:**
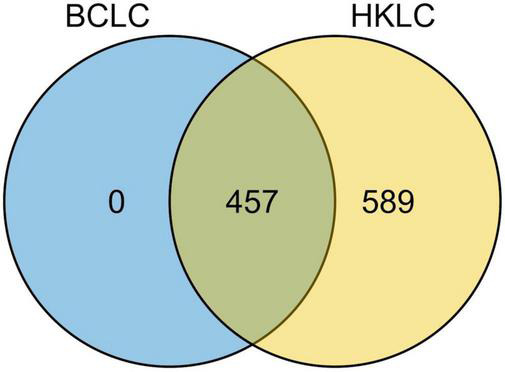
Venn diagram of the BCLC staging system and the HKLC staging system.

All the 457 IPLRs in the BCLC staging system were also IPLRs in the HKLC staging system, and the remaining 589 patients were IPLRs in the HKLC staging system but beyond the BCLC staging system (Figure 4). Compared with IPLRs in the BCLC staging system, those in the HKLC staging system but beyond the BCLC staging system had a higher percentile of HBV infection (*P* < 0.001); smaller tumor size (*P* < 0.001); more tumors (*P* < 0.001); higher frequencies of liver cirrhosis (*P* < 0.001), portal venous hypertension (*P* < 0.001), and macroscopic vascular invasion (*P* < 0.001); lower albumin level (*P* = 0.009); higher total bilirubin (*P* < 0.001), ALT (*P* < 0.001), and AST (*P* < 0.001) levels. The baseline characteristics of the patients in the two groups are compared in Table 3.

**TABLE 3:** Baseline characteristics between IPLRs in the BCLC staging and IPLRs in the HKLC staging but beyond the BCLC staging.

Characteristics	In the BCLC staging (*n* = 457)	In the HKLC staging but beyond the BCLC staging (*n* = 589)	*P*-value
Age (years, mean, SD)	54.1 ± 12.9	52.9 ± 11.2	0.080
Gender (N,%)			0.250
Male	380 (83.2)	505 (85.7)	
Female	77 (16.9)	84 (14.3)	
HBV infection (N,%)			<0.001
Positive	286 (62.6)	443 (75.2)	
Negative	171 (37.4)	146 (24.7)	
Diameter of the largest tumor (mm, median, IQR)	52 (34–80)	41 (28–65)	<0.001
Number of tumors (N,%)			<0.001
1	457 (100)	459 (77.9)	
2–3	0 (0.0)	118 (20.0)	
> 3	0 (0.0)	12 (2.0)	
Liver cirrhosis (N,%)			<0.001
Absence	279 (61.0)	211 (35.8)	
Presence	178 (39.0)	378 (64.2)	
Portal hypertension (N,%)			<0.001
Absence	457 (100.0)	226 (38.4)	
Presence	0 (0.0)	363 (61.6)	
Macroscopic vascular invasion (N,%)			<0.001
Absence	457 (100)	571 (96.9)	
Intrahepatic vascular invasion	0 (0.0)	18 (3.1)	
Creatine (mg/dL, median, IQR)	0.79 (0.69–0.91)	0.79 (0.68–0.90)	0.418
Serum albumin (g/L, median, IQR)	39.6 (37.1–42.4)	38.9 (35.9–42.1)	0.009
Total bilirubin (mg/dL, median, IQR)	0.64 (0.50–0.79)	1.03 (0.71–1.27)	<0.001
ALT (IU/L)	30.0 (19.6–45.3)	33.9 (23.7–52.8)	<0.001
AST (IU/L)	32.0 (23.7–44.9)	37.2 (27.7–55.7)	<0.001
AFP (ng/mL, N,%).			0.192
≤200	295 (64.6)	357 (60.6)	
>200	162 (35.4)	232 (39.4)	
OS (months, median, 95%CI)	Not reached	58.1 (49.7–66.5)	<0.001

IPLR, ideal patients for liver resection; HKLC, the Hong Kong Liver Cancer; BCLC, Barcelona Clinic Liver Cancer; SD, standard deviation; IQR, interquartile range; HBV, hepatitis B virus; ALT, alanine aminotransferase; AST, aspartate aminotransferase; AFP, alpha-fetoprotein; OS, overall survival; CI, confidence interval.

**FIGURE 5:**
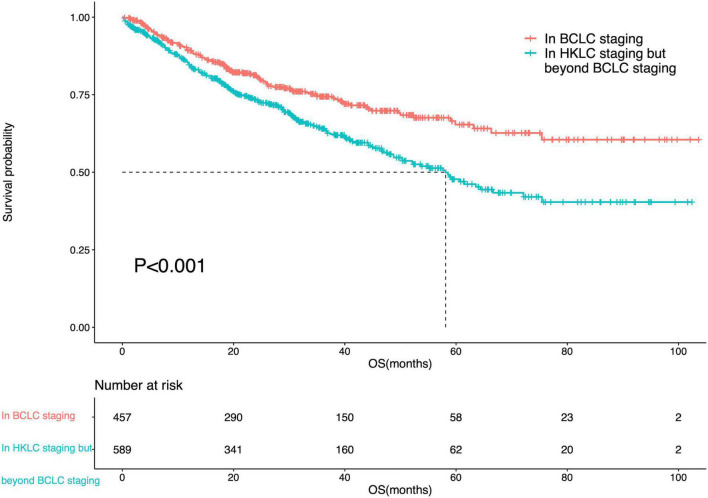
Survival curve of the IPLRs in the BCLC staging system and those in the HKLC staging system but beyond the BCLC staging system.

The median OS of IPLRs in the HKLC staging system but beyond the BCLC staging system was 58.1 months (95% CI: 49.7–66.5). There was a significant difference in OS between patients in the BCLC staging and those in the HKLC staging system but beyond the BCLC staging system (*P* < 0.001) (Figure 5).

### Risk factors of poor prognosis of ideal patients for liver resections in the Hong Kong Liver Cancer staging system but beyond the Barcelona Clinic Liver Cancer staging system

In the univariate analysis, age (*P* = 0.020), diameter of the largest tumor (*P* = 0.002), liver cirrhosis (*P* = 0.005), serum albumin (*P* = 0.028), and AST (*P* = 0.004) were influencing factors of prognosis. Therefore, these five parameters were included in the multivariate Cox regression analysis. The results showed that the diameter of the largest tumor > 5 cm (HR = 1.58; 95% CI: 1.18–2.10; *P* = 0.002) and presence of liver cirrhosis (HR = 1.61; 95% CI: 1.19–2.20; *P* = 0.002) were risk factors for poor prognosis in IPLRs in the HKLC staging system but beyond the BCLC staging system. The results of the univariate and multivariate Cox regression analyses are shown in Table 4.

**TABLE 4:** Univariate and multivariate Cox regression analysis with risk factors of poor prognosis of IPLRs in the HKLC staging system but beyond the BCLC staging system (*n* = 589).

	Univariate				Multivariate		
		
	N	HR	95% CI	P	HR	95% CI	P
Age (years)	589	1.01	(1.00–1.03)	0.020	1.01	(1.00–1.02)	0.091
Gender							
Male	505	1					
Female	84	0.80	(0.54–1.19)	0.266			
Etiology of underlying liver diseases (N)							
None	92	1					
HBV	443	1.00	(0.68–1.46)	0.980			
HCV	14	1.00	(0.41–2.36)	0.974			
Alcohol	12	1.17	(0.49–2.80)	0.724			
Mixed	28	0.80	(0.37–1.74)	0.580			
Diameter of the largest tumor (cm, N)							
≤5	366	1			1		
>5	223	1.54	(1.17–2.02)	0.002	1.58	(1.18–2.10)	0.002
Number of tumors (N)							
1	459	1					
2–3	118	1.31	(0.94–1.84)	0.115			
>3	12	1.94	(0.91–4.14)	0.086			
Liver cirrhosis (N)							
Absence	211	1			1		
Presence	378	1.54	(1.14–2.07)	0.005	1.61	(1.19–2.20)	0.002
Portal hypertension (N)							
Absence	226						
Presence	363	1.15	(0.72–1.25)	0.717			
Macroscopic vascular invasion (N)							
Absence	571	1					
Intrahepatic vascular invasion	18	1.40	(0.72–2.72)	0.330			
Serum albumin (g/L, N)							
≥35	485	1			1		
<35	104	1.45	(1.04–2.03)	0.028	1.22	(0.86–1.72)	0.270
Total bilirubin (mg/dL, N)							
≤1.0	278	1					
>1.0	311	1.15	(0.87–1.51)	0.318			
Creatine (mg/dL, N)							
≤1.2	570	1					
>1.2	19	1.75	(0.93–3.30)	0.086			
ALT (IU/L, N)							
≤40	361	1					
>40	228	1.25	(0.95–31.65)	0.110			
AST (IU/L, N)							
≤40	319	1			1		
>40	270	1.50	(1.14–1.97)	0.004	1.29	(0.97–1.72)	0.080
AFP(ng/mL, N)							
≤200	357	1					
>200	232	1.30	(0.99–1.70)	0.065			

IPLR, ideal patients for liver resection; HKLC, the Hong Kong Liver Cancer; BCLC, Barcelona clinic liver cancer; HR, hazard ratio; CI, confidence interval; HBV, hepatitis B virus; HCV, hepatitis C virus; ALT, alanine aminotransferase; AST, aspartate aminotransferase; AFP, alpha-fetoprotein.

**FIGURE 6:**
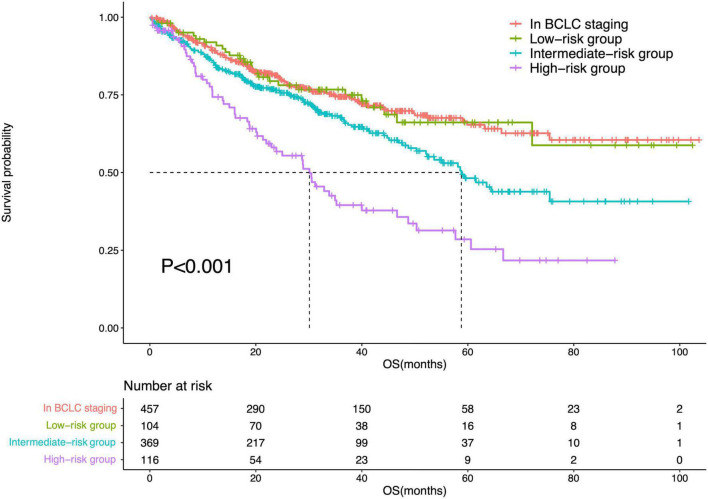
Survival curves of the IPLRs in the BCLC staging system and those in the low-, intermediate-, and high-risk groups.

The diameter of the largest tumor had a beta coefficient of 0.454, while liver cirrhosis had a beta coefficient of 0.479. As these two parameters had similar beta coefficients in the multivariate Cox regression analysis, patients were assigned one point when they had a tumor > 5 cm or liver cirrhosis. Therefore, the minimum total point was 0, while the maximum total point was 2. The 104 patients with a total score of 0 were classified into the low-risk group; 369 patients with a total point of 1, intermediate-risk group; and 116 patients with a total score of 2, high-risk group. There was a significant difference in OS among patients in the BCLC staging systems and low-risk, intermediate-risk, and high-risk groups (*P* < 0.001). Meanwhile, there was no difference in OS between patients in the BCLC staging system and those in the low-risk group (*P* = 0.996). However, differences in OS were noted between patients in the BCLC staging system and those in the intermediate-risk (*P* = 0.003) and high-risk (*P* < 0.001) groups. The survival curves are shown in Figure 6.

## Discussion

The BCLC staging system is a most popular treatment algorithm in Western countries while the HKLC staging is widely accepted in Eastern countries (14–16), and both two staging systems have their own definitions for IPLR. The 2022 version of the BCLC staging system defines IPLRs as patients with ECOG-PS score of 0, preserved liver function, single tumor regardless of size, absence of macroscopic vascular invasion and extrahepatic metastasis, and normal total bilirubin level and portal venous pressure (8). Meanwhile, in the HKLC staging system, IPLRs are defined as patients with an ECOG-PS score of 0–1, preserved liver function, absence of extrahepatic vascular invasion/metastasis, early tumor burden (≤ 5 cm, ≤ 3 tumor nodules, and absence of intrahepatic venous invasion), or intermediate tumor burden (≤ 5 cm, > 3 tumor nodules/with intrahepatic venous invasion or > 5 cm, ≤ 3 tumor nodules, and no intrahepatic venous invasion) (9). As described, the definition of IPLRs in the HKLC staging system is more aggressive than that in the BCLC staging system. In the present study, only 35.3% of patients in the entire study population were IPLRs according to the BCLC staging system, whereas the percentage was higher at 80.7% according to the HKLC staging system.

The prognostic predictive performance for OS in the BCLC staging system compared with that in the HKLC staging system has been widely investigated. A cohort study by Li et al. demonstrated that the BCLC staging system is better than the HKLC staging system in predicting survival and allocating patients to curative treatment (10). Kolly et al. also showed that the BCLC staging system offered a more accurate survival prediction than the HKLC staging system in the European population (11). However, studies by Yan et al. and de Freitas et al. showed that the HKLC staging system was more suitable for predicting prognosis in selected cases (12, 13). Apparently, it is still controversial whether one staging system is superior to the other. In the present study, IPLRs in the BCLC staging system had a better prognosis than those in the HKLC staging system (*P* = 0.011).

In the current study, all IPLRs in the BCLC staging system were also IPLRs in the HKLC staging system, as such, IPLRs in the HKLC staging system were divided into two groups, one is IPLRs in the BCLC staging system and the other is the IPLRs in the HKLC staging system but beyond the BCLC staging system. The results showed that the IPLRs in the BCLC staging system had a better survival than those in the HKLC staging system but beyond the BCLC staging system (*P* < 0.001), hence, the definition of IPLR in the HKLC staging but beyond the BCLC staging system should be carefully reconsidered. Not surprisingly, IPLRs in the HKLC staging system but beyond the BCLC staging system had higher percentile of HBV infection (*P* < 0.001); more tumors (*P* < 0.001); higher frequencies of liver cirrhosis (*P* < 0.001), portal venous hypertension (*P* < 0.001), and macroscopic vascular invasion (*P* < 0.001); lower albumin level (*P* = 0.009); higher total bilirubin (*P* < 0.001), ALT (*P* < 0.001), and AST (*P* < 0.001) levels than those in the BCLC staging system. However, interestingly, the diameter of the largest tumor is smaller in the IPLRs in the HKLC staging but beyond the BCLC staging than those in the BCLC staging. The possible explanation for this discrepancy is mainly owing to the different definitions of IPLR between these two staging systems. The tumor size is not a key element to define the IPLR in the BCLC staging system, while tumor size ≤ 5 cm is a main factor to define the IPLR in the HKLC staging system.

Although, prognosis of IPLRs in the BCLC staging system is better than that of IPLRs in the HKLC staging system as well as that of IPLRs in the HKLC staging system but beyond the BCLC staging system, the BCLC staging system is too conservative to define the IPLR and thus may exclude patients who may potentially benefit from LR. In the last decades, better patient selection and improved surgical techniques have enabled the indications for LR to be expanded. Therefore, it is essential to identify IPLRs beyond the BCLC staging system who may really benefit from LR. In the present study, univariate and multivariate Cox regression analyses showed that the diameter of the largest tumor being > 5 cm (HR = 1.58; 95% CI: 1.18–2.10; *P* = 0.002) and the presence of liver cirrhosis (HR = 1.61; 95% CI: 1.19–2.20; *P* = 0.002) were risk factors for poor prognosis in IPLRs in the HKLC staging system but beyond the BCLC staging system. Tumor size is a tumor burden-related parameter that is highly associated with the prognosis of HCC (17, 18). Liver cirrhosis is an underlying liver disease-related parameter that is widely accepted as a significant prognostic factor for survival because most HCCs derive from the background of underlying liver diseases (19, 20). These two risk factors were used to identify the population who may benefit from LR in IPLRs in the HKLC staging system but beyond the BCLC staging system. The results demonstrated that among IPLRs in the HKLC staging system but beyond the BCLC staging system, those whose largest tumor size is smaller than 5 cm and without concurrent liver cirrhosis might have comparable survival to IPLRs in the BCLC staging. This result indicates that LR can be performed in selected cases in this population.

This study had some limitations. First, the retrospective nature of the study may have led to several selection biases. Second, with approximately 70% of patients having evidence of HBV infection, our data require validation from other study groups in whom HCV infection or alcohol consumption is the prevailing etiology of chronic liver disease. Third, we only validated the performance of the two systems in patients who underwent LR. The accuracy for other treatments according to the HKLC and BCLC staging systems remains unclear. In addition, there was a lack of patients treated with liver transplantation and ablation in our cohort, which may not account for this aspect of HKLC and BCLC staging and treatment recommendations. Future studies should include more patients such as those who underwent liver transplantation and ablation to validate our findings.

## Conclusion

In conclusion, IPLRs in the BCLC staging system have better prognosis than those in the HKLC staging system and those in the HKLC staging system but beyond the BCLC staging system. This indicates that the BCLC staging system is better in identifying IPLR. However, among IPLRs in the HKLC staging system but beyond the BCLC staging system, patients with the largest tumor size measuring < 5 cm and without liver cirrhosis may obtain comparable survival benefit from LR to IPLRs in the BCLC staging system.

## Data availability statement

The original contributions presented in this study are included in the article/supplementary material, further inquiries can be directed to the corresponding author/s.

## Ethics statement

The studies involving human participants were reviewed and approved by The Second Xiangya Hospital of Central South University. Written informed consent for participation was not required for this study in accordance with the national legislation and the institutional requirements.

## Author contributions

J-XL and PZ wrote the manuscript. J-XL, PZ, W-WC, and YB provided the patients’ information. YT and YB reviewed the patients’ clinical data. D-HC, SZ, Y-DX, and W-WC edited the manuscript. W-WC revised the manuscript. Y-DX, W-WC, and SZ were the main contributors of the study design and concept. All authors contributed to the article and approved the submitted version.

## Footnotes

1
http://www.R-project.org
